# Genome-wide identification of MAPK gene family members in *Fagopyrum tataricum* and their expression during development and stress responses

**DOI:** 10.1186/s12864-022-08293-2

**Published:** 2022-02-03

**Authors:** Yingjun Yao, Haixia Zhao, Lei Sun, Wenjing Wu, Chenglei Li, Qi Wu

**Affiliations:** College of Life Science, Sichuan Agricultural University, No.46, Xinkang Road, Ya’an, 625014 Sichuan China

**Keywords:** Tartary buckwheat, MAPK, Evolution, Expression patterns, Abiotic stress, Functional verification

## Abstract

**Background:**

Mitogen-activated protein kinases (MAPKs) plays essential roles in the development, hormone regulation and abiotic stress response of plants. Nevertheless, a comprehensive study on MAPK family members has thus far not been performed in Tartary buckwheat.

**Results:**

Here, we identified 16 *FtMAPK*s in the *Fagopyrum tataricum* genome. Phylogenetic analysis showed that the *FtMAPK* family members could be classified into Groups A, B, C and D, in which A, B and C members contain a Thr-Glu-Tyr (TEY) signature motif and Group D members contain a Thr-Asp-Tyr (TDY) signature motif. Promoter *cis*-acting elements showed that most *Pro*_*FtMAPks*_ contain light response elements, hormone response elements and abiotic stress response elements, and several *Pro*_*FtMAPks*_ have MYB-binding sites, which may be involved in the regulation of flavonoid biosynthesis-related enzyme gene expression. Synteny analysis indicated that *FtMAPK*s have a variety of biological functions. Protein interaction prediction suggested that MAPKs can interact with proteins involved in development and stress resistance. Correlation analysis further confirmed that most of the *FtMAPK* genes and transcription factors involved in the stress response have the same expression pattern. The transient transformation of *FtMAPK1* significantly increased the antioxidant enzymes activity in Tartary buckwheat leaves. In addition, we also found that *FtMAPK1* can respond to salt stress by up-regulating the transcription abundance of downstream genes.

**Conclusions:**

A total of 16 *MAPK*s were identified in Tartary buckwheat, and the members of the MAPK family containing the TDY motif were found to have expanded. The same subfamily members have relatively conserved gene structures and similar protein motifs. Tissue-specific expression indicated that the expression of all *FtMAPK* genes varied widely in the roots, stems, leaves and flowers. Most *FtMAPK*s can regulate the expression of other transcription factors and participate in the abiotic stress response. Our findings comprehensively revealed the *FtMAPK* gene family and laid a theoretical foundation for the functional characterization of *FtMAPKs*.

**Supplementary Information:**

The online version contains supplementary material available at 10.1186/s12864-022-08293-2.

## Background

To respond to various biotic and abiotic threats, plants have evolved a series of sophisticated signalling networks to manage external stresses. MAPK cascades play critical roles in the response to external stimuli and represent one of the primary mechanisms controlling signal transduction. The MAPK cascade pathway transmits external stimulus signals to cells, after which the organism realizes the regulation of response to the stimulus through the phosphorylation and dephosphorylation of proteins.

MAPK signal transduction modules are traditionally composed of three sequentially activated kinases. In general, external signals can activate MAPKKKs, which phosphorylate downstream MAPKKs, while phosphorylated MAPKKs ultimately phosphorylate MAPKs [1]. Activated MAPKs eventually phosphorylate various downstream transcription factors and other signalling components [2].

MAPKs function towards the end MAPK cascade pathways and contain 11 conserved protein kinase motifs. There is a TXY motif between domains VII and VII [3], and phosphorylated TXY is essential for activating MAPK. Plant MAPKs can be separated into Groups A, B, C, and D. Members of Groups A, B, and C contain the characteristic Thr-Glu-Tyr (TEY) motif at their phosphorylation site. However, members of Group D possess a Thr-Asp-Tyr (TDY) motif. Due to the importance of MAPKs in coping with adverse conditions, the MAPK gene family has been studied in depth: members of this family have been identified and reported in various plant species and present quite complex features. To date, there are 20, 15, 19, 19, 18, 20, 28, and 54 *MAPK*s in *Arabidopsis* [2], rice [4], maize [5], chickpea [6], *Actinidia chinensis* [7], barley [8], *Gossypium raimondii* [9] and bread wheat [10]. Similarly, 12 candidate *MAPK* genes have been identified in *Fragaria vesca* [11]. With an improved in-depth understanding of *MAPK*, the function of *MAPK* has attracted widespread attention and research.

A large number of studies have confirmed that *MAPKs* participate in a variety of biological functions. The expression levels of all *MAPK*s are increased significantly under abiotic stress and abscisic acid (ABA) treatment in chickpea [6]. Overexpression of *OsMAPK33* weakened the tolerance to salt stress in rice [12]. Under cold treatment, the expression of *MAPKs* increased significantly in *Jatropha curcas*. *MAPK*s are also involved in the growth and development of plants. *AtMAPK6* kinase is involved in regulating many aspects of plant development, including the development of anthers, inflorescences, and embryos [13]. In addition, *MAPK*s are involved in response to hormones. Specifically, several *MAPK*s are involved in the ethylene response process during the ripening of bananas. Moreover, MAPK can interact with other transcription factors in response to adversity. *OsWRKY55* interacts with four MAPKs that could be induced by drought: *OsMPK7*, *OsMPK9*, *OsMPK20-1*, and *OsMPK20-4* [14]. *MdMYB1* can be phosphorylated by MdMPK4 to enhance light-induced anthocyanin accumulation in apples [15]. Therefore, MAPKs confer essential biological functions directly or in combination with transcription factors to plants.

Tartary buckwheat, a grain crop species used for both food and medicine, has attracted much attention because of its rich content of flavonoids. Because Tartary buckwheat has good adaptability to drought, salt, high temperature/low temperature; etc., it is crucial to reveal stress resistance mechanisms and identify stress resistance-related gene families. The MAPK cascade is an essential aspect of studying the mechanism of resistance to stress in plants. We conducted comprehensive research, including a phylogenetic, chromosome localization, gene structure, protein motif, *cis*-acting element, evolutionary, expression profile, protein interaction, correlation analysis and functional verification. Overall, a comprehensive study of the *FtMAPK* gene family and the expression pattern of *FtMAPK* in response to abiotic stress have laid a foundation for the functional characterization and expression regulation of *FtMAPK* in Tartary buckwheat.

## Results

### Identification of MAPK family genes in Tartary Buckwheat

To fully understand the evolutionary history of the *MAPK* family of Tartary buckwheat and its importance in abiotic stress, a total of 16 MAPK genes were identified in Tartary buckwheat, designated *FtMAPK1-FtMAPK16* (Additional file 1). All MAPKs in Tartary buckwheat contained the phosphorylation sites TEY or TDY by Batch-CD Search (https://www.ncbi.nlm.nih.gov/Structure/bwrpsb/bwrpsb.cgi) analysis and TBtools visualization (Additional file 2).

In addition, we analysed the characteristics of the 16 *FtMAPK*s in *Fagopyrum tataricum* (Table 1). Briefly, the protein length ranged from 371(MAPK6) to 609 (MAPK16) amino acids. The protein molecular weight (MW) ranged from 42.6 kDa (MAPK6) to 69.4 kDa (MAPK16), and the isoelectric point (pI) varied from 5.39 (MAPK4) to 9.25 (MAPK11). Subcellular localization prediction revealed that 8 MAPK proteins were located in the cytoplasm, 3 MAPK proteins were located in the microbody, 1 MAPK protein was found in the chloroplast stroma, and the other proteins were located in the nucleus. However, none of the MAPK proteins have a transmembrane domain. Only two MAPK proteins (MAPK4 and MAPK14) have signal peptides, and none of the other MAPK proteins have signal peptides (Table 1).

**Table 1:** Characteristics of 16 the *FtMAPKs* in Tartary buckwheat

Gene name	Gene ID	Protein length (aa)	MW	pI	Subcellular location	Transmembrane domain	Signal peptide
FtMAPK1	FtPinG0006545900.01	385	44.2	6.86	M	0	NO
FtMAPK2	FtPinG0004152000.01	388	44.2	6.19	N	0	NO
FtMAPK3	FtPinG0005197700.01	407	46.7	5.43	C	0	NO
FtMAPK4	FtPinG0000626000.01	372	43.0	5.39	N	0	YES
FtMAPK5	FtPinG0004095200.01	406	46.4	6.90	CS	0	NO
FtMAPK6	FtPinG0001815800.01	371	42.6	5.41	C	0	NO
FtMAPK7	FtPinG0006454600.01	381	44.3	5.66	C	0	NO
FtMAPK8	FtPinG0008104600.01	452	51.2	8.34	C	0	NO
FtMAPK9	FtPinG0005315200.01	567	64.7	8.84	N	0	NO
FtMAPK10	FtPinG0009258900.01	608	68.9	9.40	C	0	NO
FtMAPK11	FtPinG0005630300.01	589	66.9	9.25	C	0	NO
FtMAPK12	FtPinG0005324100.01	405	47.1	8.93	M	0	NO
FtMAPK13	FtPinG0003584300.01	570	64.6	9.33	M	0	NO
FtMAPK14	FtPinG0009311600.01	543	61.8	6.51	C	0	YES
FtMAPK15	FtPinG0009346800.01	571	65.5	7.41	C	0	NO
FtMAPK16	FtPinG0001873100.01	609	69.4	9.47	N	0	NO

*MW* molecular weight, *pI* isoelectric points, *M* microbody, *N* nucleus, *C* cytoplasm, *CS* chloroplast stroma

### Phylogenetic analyses of *FtMAPK* genes

**Fig. 1:**
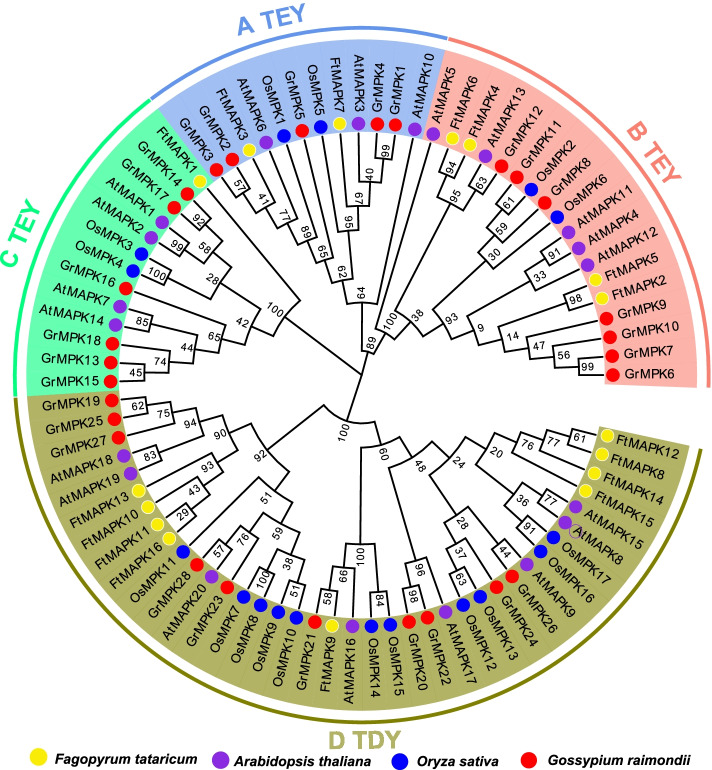
Phylogenetic relationship of MAPK genes in *F. tataricum*, Ara*bidopsis, O.sativa* and *G.raimondii*. A TEY, B TEY, C TEY and D TDY exhibit different gene clusters. The full-length MAPK protein sequences were aligned using the MUSCLE tool, and the phylogenetic tree was constructed using MEGA 7.0.26 by the NJ method with default parameters and 1000 bootstrap replicates. Finally, the tree was visualized with the online tool EvolView (https://evolgenius.info//evolview-v2/#mytrees/1/2)

To investigate the evolutionary relationships among the MAPK proteins, a phylogenetic tree was constructed with amino acid sequences of 16 putative *FtMAPK*s from Tartary buckwheat, 20 *AtMAPK*s from *Arabidopsis*, 15 *OsMAPK*s from *Oryza sativa* and 28 *GrMAPK*s from *Gossypium raimondii* (Fig. 1). The results showed that the 16 *FtMAPK*s could be divided into Groups A, B, C and D. Among them, *FtMAPK*s belonging to Groups A, B, and C have a TEY motif, whereas *FtMAPK*s classified into Group D possess a TDY motif. *FtMAPK3* and *FtMAPK7* were assigned to Cluster A; *FtMAPK2*, *FtMAPK4*, *FtMAPK5* and *FtMAPK6* were assigned to Cluster B; and *FtMAPK1* clustered into Cluster C. However, *FtMAPK8*, *FtMAPK9*, *FtMAPK10*, *FtMAPK11*, *FtMAPK12*, *FtMAPK13*, *FtMAPK14*, *FtMAPK15*, and *FtMAPK16* were assigned to Cluster D (Fig. 1). Interestingly, the *FtMAPK*s in Group D had significantly expanded, as 9 *FtMAPK*s were present in Tartary buckwheat. Moreover, the other 3 species also had the most members in Group D, and the expansion of Group D genes provides a reference for the evolution of Tartary buckwheat. Compared with group D, Group C included only *FtMAPK1*, indicating that the members of Group C had significantly contracted. We speculate that *FtMAPK1* is very important for the study of the function of Tartary buckwheat.

### Chromosomal location of *FtMAPK*s in the Tartary buckwheat genome

**Fig. 2:**
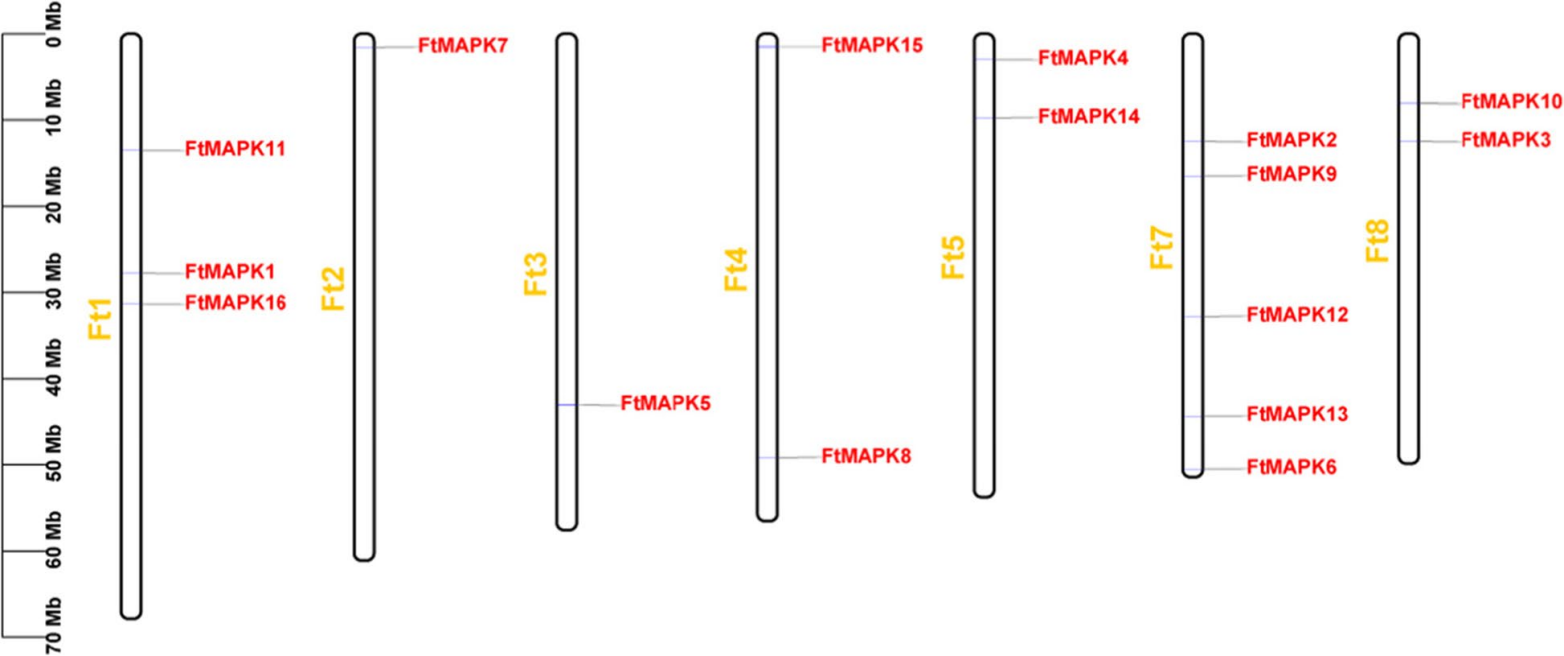
Schematic representations of the chromosomal distributions of the *FtMAPK* genes. The vertical bars mark the chromosomes of Tartary buckwheat. The chromosome number is located to the left of each chromosome. The scale on the left represents the chromosome length

Chromosome mapping analysis revealed that 16 *FtMAPK* genes were present on all chromosomes except chromosome 6. The results showed that chromosomes Ft2 and Ft3 have only one *FtMAPK*, *FtMAPK7* and *FtMAPK5*, respectively. In addition, two *FtMAPK* genes were found on chromosomes Ft4, Ft5, and Ft8. In contrast, half of the *FtMAPK* genes were distributed on chromosomes Ft1 and Ft7 (Fig. 2). Furthermore, *FtMAPK* presents an uneven distribution, and no tandem repeat genes appear. Remarkably, *FtMAPK*s belonging to the same group in the phylogenetic tree are not mapped on the same chromosome, but are scattered in different positions on the same chromosome, or exist on different chromosomes. This shows that tandem duplication events did not play an important role during the expansion of the *FtMAPK* family.

### Gene structure and motif composition of MAPKs in Tartary buckwheat

**Fig. 3:**
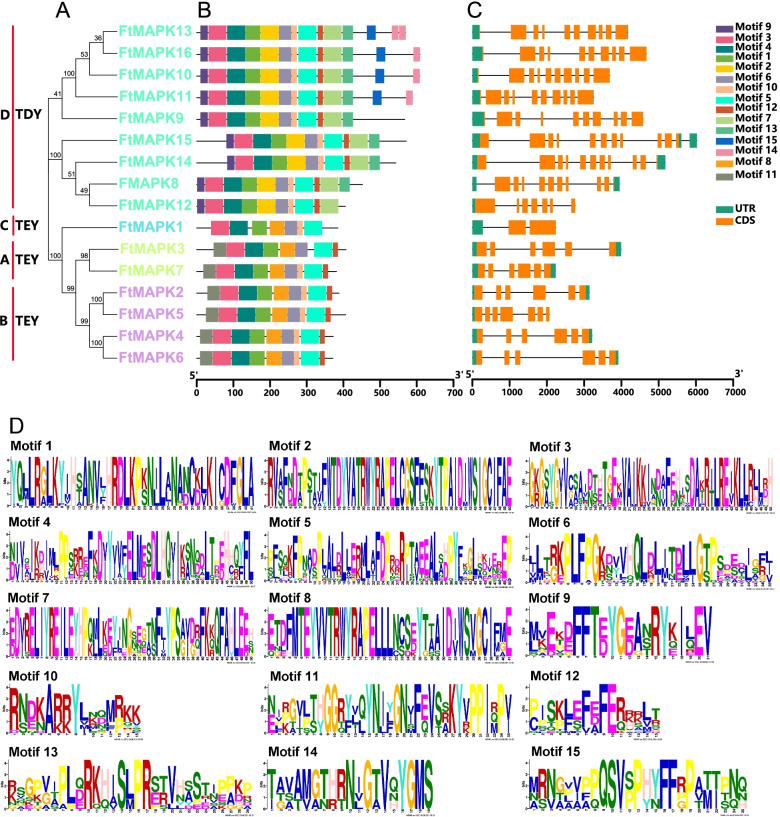
Phylogenetic relationships, conserved protein motifs and gene structures of the 16 *FtMAPKs*. **A** The phylogenetic tree was constructed based on the full-length sequences of Tartary buckwheat MAPK proteins using MEGA 7.0.26 and was visualized by the online tool EvolView (https://evolgenius.info//evolview-v2/#mytrees/1/2). Yellow–green represents the A TEY gene cluster. Purple represents the B TEY gene cluster. Blue represents the C TEY gene cluster. Green represents the D TEY gene cluster. **B** Motifs distribution of the MAPK proteins. The conserved motifs of MAPK proteins were determined by MEME (http://meme-suite.org/tools/meme) and visualized by TBtools v1.098669. The motifs, numbered 1–15, are displayed in different coloured boxes. **C** Exon-intron structures of the *FtMAPKs* genes. Orange boxes indicate exons; black lines indicate introns. **D** Sequence logo of the MAPK proteins motifs. The height of each amino acid represents the relative frequency of the amino acid at that position

A phylogenetic tree was constructed from 16 amino acid sequences of FtMAPK, and the gene family was divided into four groups. Group D is the largest with 9 members, but group C has only one member *FtMAPK1* (Fig. 3A). We analysed the conserved motifs of MAPKs to explore the various biological functions of the protein domains. Fifteen conserved motifs were identified, and each motif is present (Figs. 3B and D). The 16 identified MAPK proteins contained motif 2 (which contains the TDY signature motif) or motif 8 (which contains the TEY signature motif), indicating that all *FtMAPK*s belong to the *MAPK* gene family. Specifically, the members in the same subfamily shared similar conserved motifs. Nine MAPKs belonging to Group D contained TDY characteristic motifs, and the MAPKs classified into Groups A, B and C contained characteristic TEY motifs. N-terminal motif 9 of MAPK in Group D also contains the TDY/TEY motif, but it is not particularly conserved. Additionally, the FtMAPK of Group D includes specific motif 9 at the N-terminus and specific motifs 7, 13, 14 and 15 at the C-terminus. Whether the differences in motif composition between the members of Group D and the members of Groups A, B and C affect their biological functions, needs to be further investigated through biological experiments (Fig. 3B).

We also investigated the exon-intron pattern of the 16 *FtMAPK*s (Fig. 3C). The results showed that *FtMAPK*s in Group A exhibit conservation of their exon-intron distribution. The two *FtMAPK*s with the closest relationship in Group B had the same intron-exon distribution pattern. However, *FtMAPK1* in Group C includes only two exons-introns. The structures of *FtMAPK*s in Groups A, B and C were more conserved than were those of the *FtMAPK*s in Group D, showing a complex distribution of exons and introns. *FtMAPK12* has seven exons, *FtMAPK8*, *FtMAPK9*, *FtMAPK14* and *FtMAPK15* have 11 exons, and the other *FtMAPK*s consist of 13 exons.

### Analysis of *FtMAPK*s promoter

**Fig. 4:**
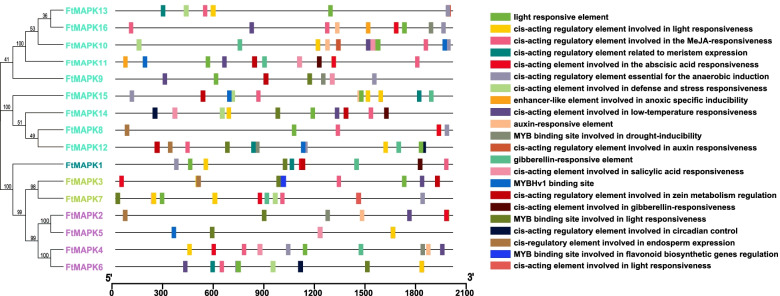
*Cis*-acting elements in the promoter regions of 16 *FtMAPKs*. *Cis*-elements with similar functions are displayed in the same colour. The black line indicates the promoter length of the *FtMAPK* genes. The different coloured boxes on the right represent *cis*-acting elements with different functions

To better understand the function of *FtMAPK*s and the precise regulation of stress-responsive gene expression, the *cis*-acting elements in the promoter region of *FtMAPK*s were identified and analysed (Fig. 4; Additional file 3). The results suggested that most *FtMAPK*s contain hormone (methyl jasmonate [MeJA] and ABA) responsive elements. All *Pro*_*FtMAPK*__s_ contain light-responsive elements. The promoters of 7 *FtMAPK*s (*FtMAPK1*, *FtMAPK3*, *FtMAPK9*, *FtMAPK11*, *FtMAPK12*, *FtMAPK14* and *FtMAPK15*) contain regulatory elements (O2 sites) involved in zein metabolism regulation. A few *FtMAPK*s have CAT boxes and circadian function engaged in endosperm expression and circadian control, respectively. The *cis*-acting elements in the promoters of other *FtMAPK*s are also involved in the abiotic stress response, including *6 Pro*_*FtMAPK*__s_ (*FtMAPK6*, *FtMAPK7*, *FtMAPK10*, *FtMAPK13*, *FtMAPK14* and *FtMAPK15*) containing *cis*-regulating elements involved in defence and stress responsiveness, and 9 *Pro*_*FtMAPK*__s_ (*FtMAPK2*, *FtMAPK3*, *FtMAPK4*, *FtMAPK6*, *FtMAPK9*, *FtMAPK10*, *FtMAPK11*, *FtMAPK14* and *FtMAPK16*) containing *cis*-regulating elements involved in low-temperature responsiveness. There are even several *Pro*_*FtMAPK*__s_ (*FtMAPK2*, *FtMAPK4*, *FtMAPK9*, *FtMAPK12* and *FtMAPK16*) that combine with MYB transcription factors to participate in the drought response. In addition, there are three *Pro*_*FtMAPK*__s_ (*FtMAPK3*, *FtMAPK10* and *FtMAPK16*) that can be combined with MYB to participate in the regulation of flavonoid biosynthesis. There are also *cis*-regulatory elements involved in the expression of meristems (Fig. 4). These results illustrate the potential role of *FtMAPK*s in plant growth and development, the response to stress and hormone signalling pathways.

### Tertiary structure of MAPK proteins in Tartary buckwheat

**Fig. 5:**
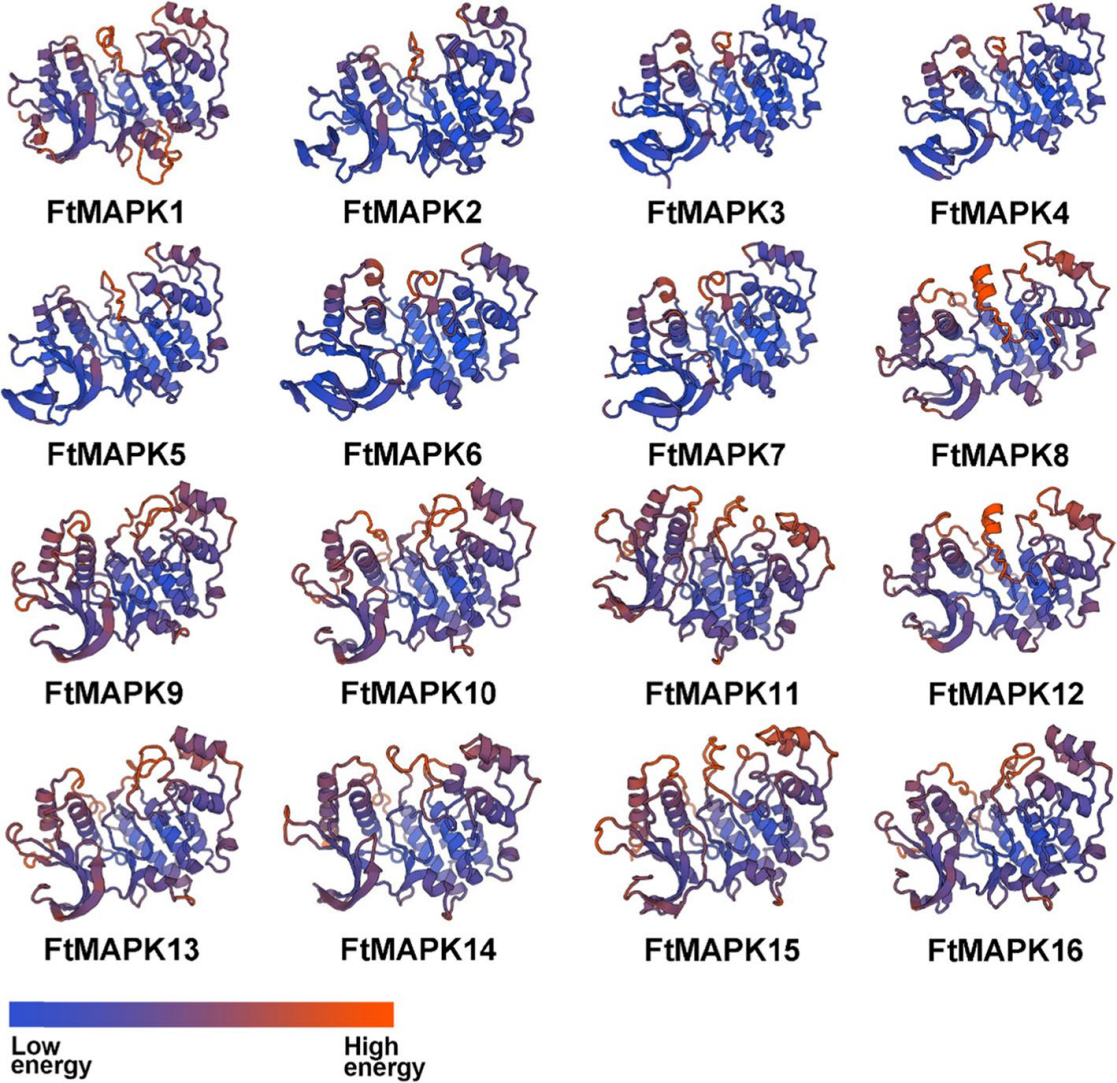
Predicted three-dimensional domains of MAPK proteins from *Fagopyrum tataricum*. 3D models of the 16 MAPKs according to SWISS-MODEL. The blue colour indicates a low-energy structure, and the orange colour indicates a high-energy structure

The tertiary structures of MAPK proteins are shown in Fig. 5, and they are mainly composed of α-helices, β-folds and random coils. MAPK1, MAPK2, MAPK3, MAPK4, MAPK5, MAPK6, MAPK7, MAPK8 and MAPK12 have identical structures, indicating that they have similar functions. In addition, MAPK9, MAPK10, MAPK13 and MAPK16 are structurally similar, as are MAPK11, MAPK14 and MAPK15 (Fig. 5). It is speculated that proteins with different structures may determine the diversity of *FtMAPK* functions.

### Gene duplication and synteny analysis of the *FtMAPK* gene families

**Fig. 6:**
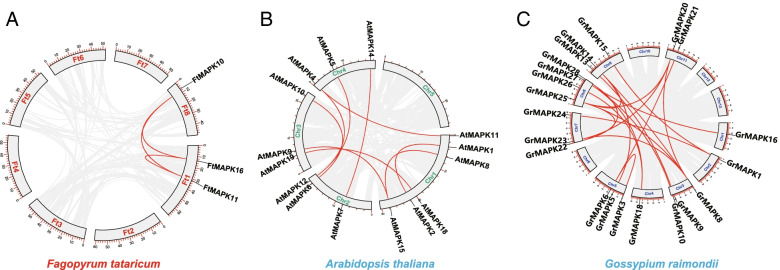
Schematic representations of the interchromosomal relationships of the *MAPK* genes from different plants. **A** The relationship between chromosomes of *FtMAPKs* from *Fagopyrum tataricum* was determined by using multiple collinear scanning toolkits (MCScanX) and was visualized by TBtools v1.098669. Grey lines represent the collinear blocks within the Tartary buckwheat genome, while the red lines highlight the syntenic *MAPK* gene pairs. **B** The relationship between chromosomes of the *AtMAPKs* from *A. thaliana* was determined by using multiple collinear scanning toolkits (MCScanX) and was visualized by TBtools v1.098669. Grey lines represent the collinear blocks within the *A. thaliana* genome, while the red lines highlight the syntenic *MAPK* gene pairs. **C** The relationship between chromosomes of the *GrMAPKs* from *G. raimondii* was determined by using multiple collinear scanning toolkits (MCScanX) and was visualized by TBtools v1.098669. Grey lines represent the collinear blocks within the *G. raimondii* genome, while the red lines highlight the syntenic *MAPK* gene pairs. The number of chromosomes is displayed in the middle of each chromosome

There are three pairs of segmental duplications on the chromosomes of Tartary buckwheat, and they are present only on chromosomes 1 and 8 (Fig. 6A). We speculate that the degree of evolution of *FtMAPK*s is relatively conservative within the species. To further understand the replication event of the *FtMAPK* genes, the replication events of *MAPK* were compared between Tartary buckwheat and other species (*Arabidopsis thaliana* and *Gossypium raimondii*). These results also demonstrated that segmental duplication *MAPK* gene pairs were found in the genomes of *Arabidopsis* (9 pairs) and *Gossypium raimondii* (19 pairs) (Fig. 6B-C).

**Fig. 7:**
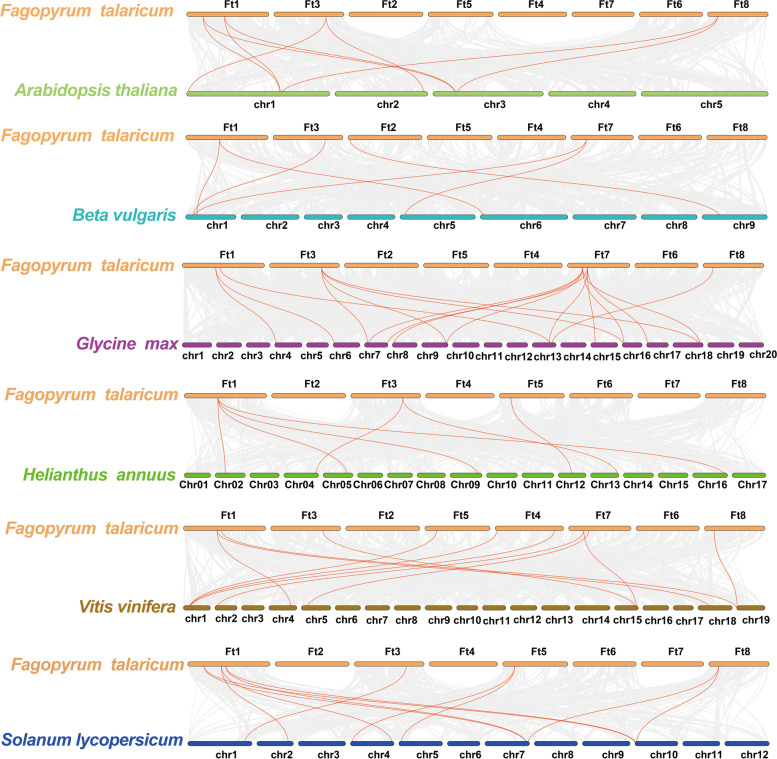
Collinearity analysis of *MAPK* genes between *F. tataricum* and 6 other plants. Grey lines indicate collinear blocks within the *F. tataricum* genome and other plant genomes, and the red curve indicates *MAPK* genes with collinearity

The synteny between species provides insight for studying the evolution of gene families and gene function. Therefore, we analysed the collinearity of *MAPK* between Tartary buckwheat and six other plants (Fig. 7). Collinearity analysis showed that the homologous genes between *Fagopyrum tataricum* and *Glycine max* were the most common, with 17 homologous gene pairs, followed by *Vitis vinifera* (11 homologous gene pairs), *Solanum lycopersicum* (11 homologous gene pairs), *Arabidopsis thaliana* (8 homologous gene pairs), *Helianthus annuus* (7 homologous gene pairs) and *Beta vulgaris* (6 homologous gene pairs). In the collinearity analysis between Tartary buckwheat and soybean, *FtMAPK2* had homologous pairs with five *MAPK* genes in soybean, indicating that *FtMAPK2* may play an essential role in the evolution of the Tartary buckwheat *MAPK* family. These results revealed that the correlation between *FtMAPK* genes and *GmMAPK* genes is similar, which is significant for exploring the relationships among species and forecasting gene function.

### Expression analysis of *FtMAPK* genes in different tissues

**Fig. 8:**
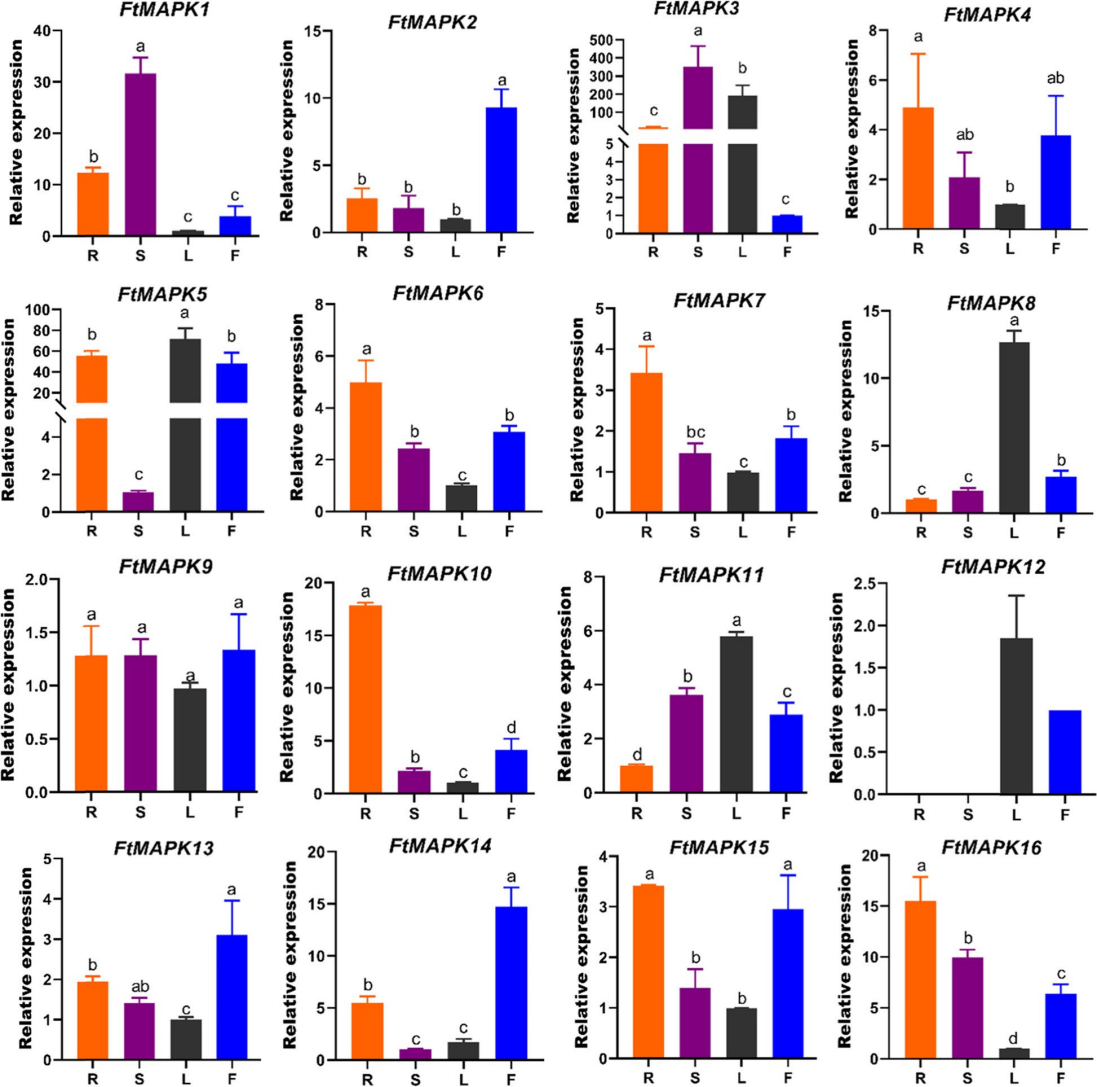
qRT–PCR analysis of *FtMAPKs* in different tissues. R (root), S (stem), L (leaf), F (flower). The lowest expression level of all genes in the four tissues was set as 1. The error bars were obtained from three measurements. Letter(s) above the bar represent significant differences (α= 0.05, Duncan) among different tissues

Exploring tissue-specific expression profiles provides a basis for studying gene functions. Therefore, we investigated the tissue-specific expression of *FtMAPK*s*.* The results showed that the expression levels of 16 *FtMAPK* genes in the roots, stems, leaves and flowers of Tartary buckwheat varied widely (Fig. 8). For instance, most *FtMAPK*s are ubiquitously expressed in every tissue of Tartary buckwheat, but *FtMAPK12* is expressed only in leaves and flowers. *FtMAPK3* had the highest expression in stems and leaves, and *FtMAPK5* was predominantly expressed in roots, leaves and flowers compared with the other tissues; in contrast, *FtMAPK4*, *FtMAPK6*, *FtMAPK7*, *FtMAPK9*, *FtMAPK11*, *FtMAPK13* and *FtMAPK15* had lower expression levels in all tissues (Fig. 8).

### Expression of *FtMAPK* genes under different stress and hormone treatments

**Fig. 9:**
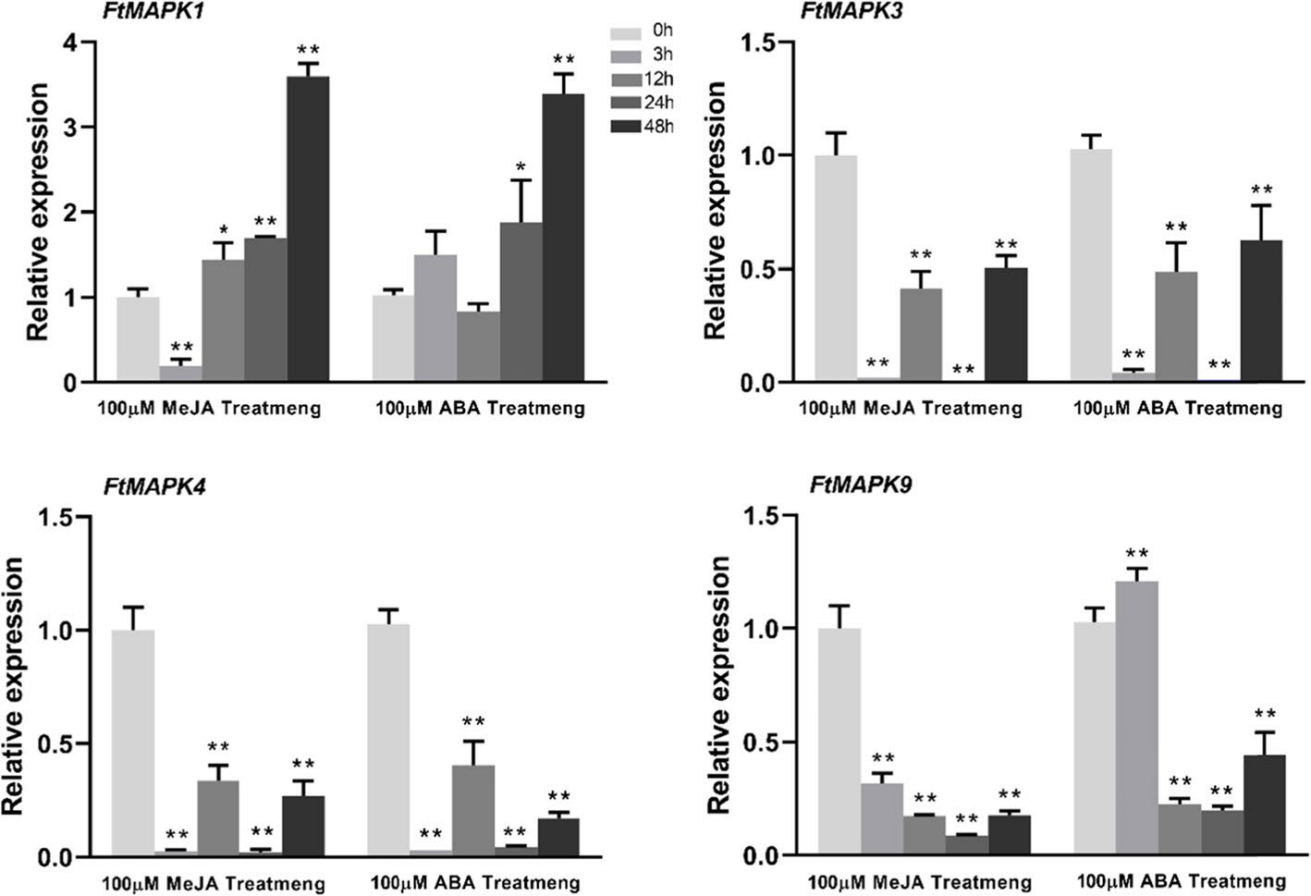
Relative expression of *FtMAPK* genes under MeJA and ABA treatments. The expression of *FtMAPK* genes at 0 h in the dark was set to “1”. The Values are the means ± SDs (*n* = 3). The Bars represent the standard deviations of three independent experiments. The asterisks indicate significant differences (*P< 0.05; **P<0.01)

Analysis of *cis*-acting elements indicated that most of the *Pro*_*FtMAPK*__s_ contained MeJA and ABA response elements. We randomly selected a *FtMAPK* from each subfamily. To preliminarily investigate the potential role of MAPKs under hormone treatment (MeJA and ABA), we determined the expression pattern of *FtMAPK*s by qRT–PCR (Fig. 9). Interestingly, the transcript levels of *FtMAPK1* were upregulated in response to hormone processing and reached the highest levels at 48 h. However, the above two hormone treatments downregulated the expression of the other three genes (*FtMAPK3, FtMAPK4* and *FtMAPK9*) compared with their expression before treatment. ABA treatment induced *FtMAPK1* and *FtMAPK9* to reach their highest levels at 48 h and 3 h, respectively. Compared with before treatment, the expression of *FtMAPK3* and *FtMAPK4* was downregulated.

**Fig. 10:**
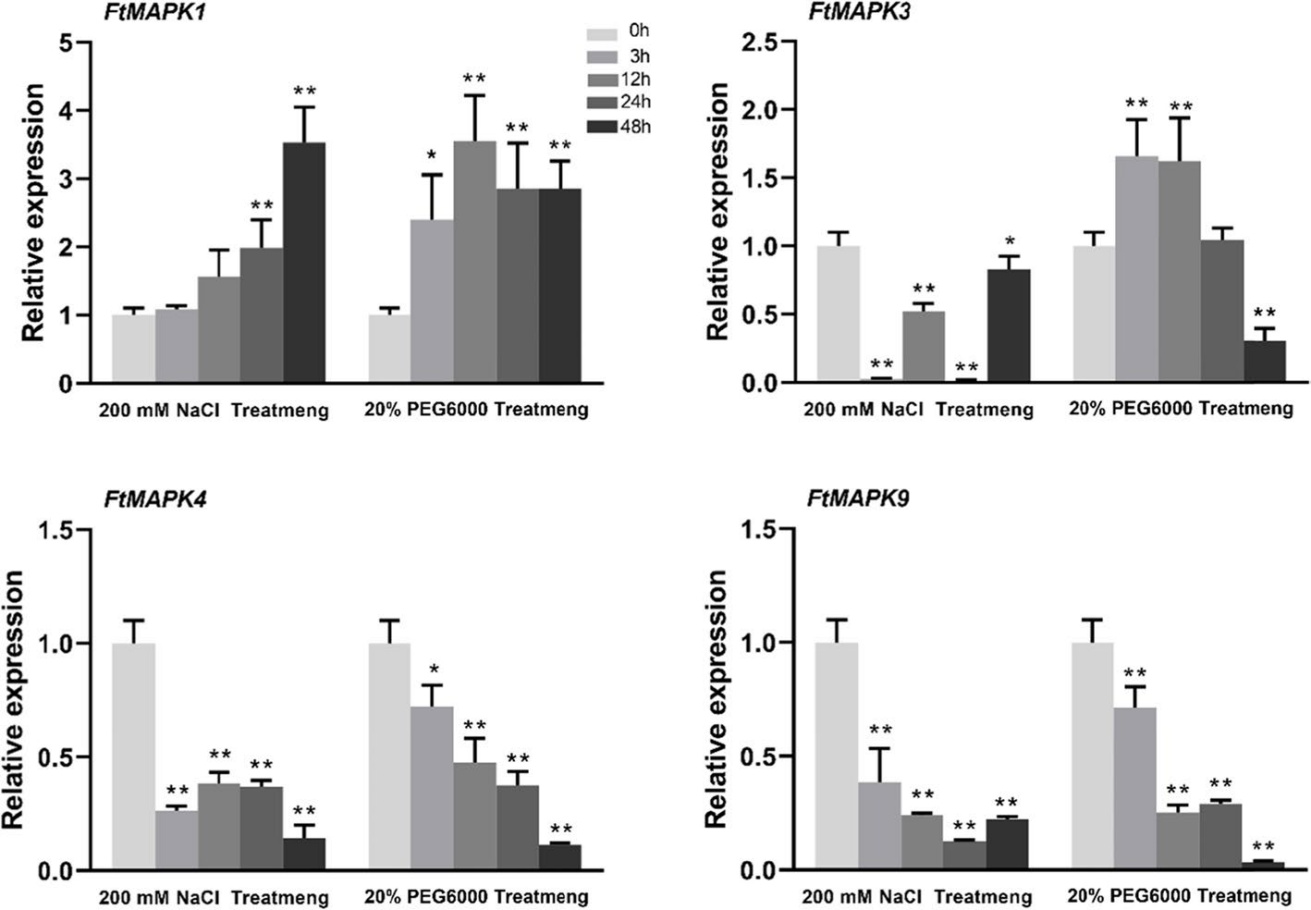
Relative expression of *FtMAPK* genes under NaCl and PEG6000 treatments. The expression of *FtMAPK* genes at 0 h in the dark was set to “1”. The bars represent the standard deviations of three independent experiments. The asterisks indicate significant differences (*P< 0.05; **P<0.01)

We also investigated the role of *FtMAPK*s in the response to drought and salt stress (Fig. 10). The expression level of *FtMAPK1* was significantly upregulated throughout the treatment process and reached its highest level at 48 h, whereas the *FtMAPK3*, *FtMAPK4* and *FtMAPK9* genes were downregulated after salt treatment. With drought treatment, the expression levels of *FtMAPK1* and *FtMAPK3* were significantly upregulated and peaked at 12 h and 3 h, respectively. However, the expression levels of *FtMAPK4* and *FtMAPK9* were downregulated at all treatment time points (Fig. 10). The downregulation of gene expression seems to indicate that the activation of certain protein kinase activities may not be related to their transcriptional levels under stress.

### Prediction and correlation analysis of MAPK interacting proteins

**Fig. 11:**
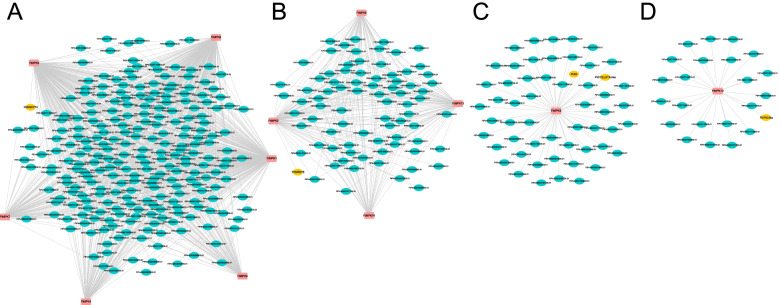
Functional interaction network analysis of FtMAPK cascade proteins. **A** The proteins with red background are FtMAPK1, FtMAPK4, FtMAPK5 and FtMAPK7–9. The protein with a yellow background is FtWRKY74; **B** The proteins with a red background are FtMAPK2, FtMAPK6 and FtMAPK13–14. **C** The protein with a red background is FtMAPK3. The proteins with yellow backgrounds are FtAG and PISTILLATA-like; **D** The protein with a red background is FtMAPK12. The protein with a yellow background is FtHY5-like, and the gene function on the blue background above has not been identified

The construction of protein interaction networks is significant for studying gene interactions and regulatory relationships. Here, to identify proteins that interact with MAPK in Tartary buckwheat, an interactive network involving MAPK1-9 and MAPK12-14 was created. The results show that MAPK5 can interact with WRKY74. Therefore, we speculate that *MAPK5* regulates the expression of *FtWRKY74* and ultimately responds to external stress (Fig. 11A). In addition, the interactive protein *FtWRKY6* involved in the *FtMAPK2* network can respond to salt, drought, cold and heat stress (Fig. 11B). *FtAG* and *FtPISTILLATA* are involved in the development of shelling and floral organs. We speculated that the interactive proteins AG and PISTILLATA-like participate in the MAPK3 network and regulate the development of Tartary buckwheat by interacting with MAPK3 (Fig. 11C). Additionally, *FtMAPK12* regulates the expression of *FtHY5-like* (Fig. 11D). It has been reported that *FtHY5* not only participates in the biosynthesis of anthocyanins but also participates in plant growth and development and response to stress [16–21]. These results preliminarily indicate that *FtMAPK*s participate in growth and development and severe vital roles in abiotic stress in plants.

**Fig. 12:**
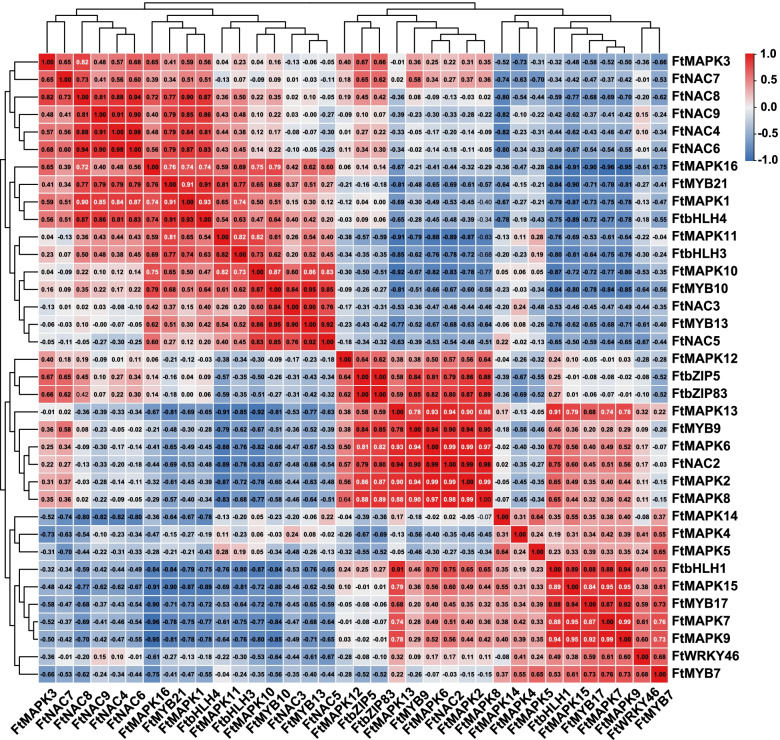
Correlations of expression patterns between Tartary buckwheat *FtMAPK* genes and other transcription factors was visualized by RStudio. Positive number: positively correlated; negative number: negatively correlated. White numbers indicate a significant correlation at the 0.01 level. *FtMYB7* (*FtPinG0003734600.01*), *FtMYB9* (*FtPinG0002001900.01*), *FtMYB10* (*FtPinG0002706600.01*), *FtMYB1 3*(*FtPinG0005410000.01*), *FtMYB17* (*FtPinG0006925500.01*), *FtMYB21* (*FtPinG0004929500.01*), *FtWRKY46* (*FtPinG0003025100.01*), *FtNAC2* (*FtPinG0005692100.01*), *FtNAC3* (*FtPinG0000381200.01*), *FtNAC4* (*FtPinG0005791100.01*), *FtNAC5* (*FtPinG0006190400.01*), *FtNAC6* (*FtPinG0005624400.01*), *FtNAC7* (*FtPinG0005167000.01*), *FtNAC8* (*FtPinG0002252000.01*), *FtNAC9* (*FtPinG0002967400.01*), *FtbHLH1* (*FtPinG0005249800.01*), *FtbHLH3* (GenBank:KU296217.1), *FtbHLH4* (*FtPinG0002267300.01*), *FtbZIP5* (*FtPinG0003196200.01*), *FtbZIP83* (*FtPinG0002143600.01*)

To further investigate whether *FtMAPK*s are involved in drought or salt stress, we analysed the correlation between *FtMAPK*s and other transcription factors involved in the abiotic stress response (Fig. 12). The results showed a positive correlation between the expression levels of *FtMAPK1* and *FtNAC8*, *FtNAC9*, *FtNAC4*, *FtNAC6*, *FtMYB21* and *FtbHLH4*. We further confirmed that *FtMAPK1* is involved in the abiotic stress response process. The correlation between *FtMAPK4* and other transcription factors was significantly reduced. FtMAPK4 was negatively correlated with the expression level of most transcription factors compared with the correlation between *FtMAPK1* and other transcription factors. There was a significant positive correlation among the expression levels of *FtMAPK9*, *FtbHLH1* and *FtMYB17*. These results show that *FtMAPK9* has the same expression pattern as *FtbHLH1* and *FtMYB17*. In addition, there was a positive correlation among four *FtMAPK*s (*FtMAPK2*, *FtMAPK6*, *FtMAPK8* and *FtMAPK13*) with respect to *FtNAC2* and *FtMYB9*. There was a significant positive correlation between *FtMAPK10* with respect to *FtMYB10*, *FtMYB13* and *FtNAC5*. There was a significant positive correlation between *FtMAPK11* and *FtbHLH13. FtMAPK15*, *FtMAPK7* and *FtMAPK9* had significant positive correlations with *FtbHLH1* and *FtMYB17*. In summary, the correlation analysis shows that most *FtMAPK*s participate in the stress response.

### Effect of overexpression of *FtMAPK1* on salt tolerance of Tartary buckwheat

**Fig. 13:**
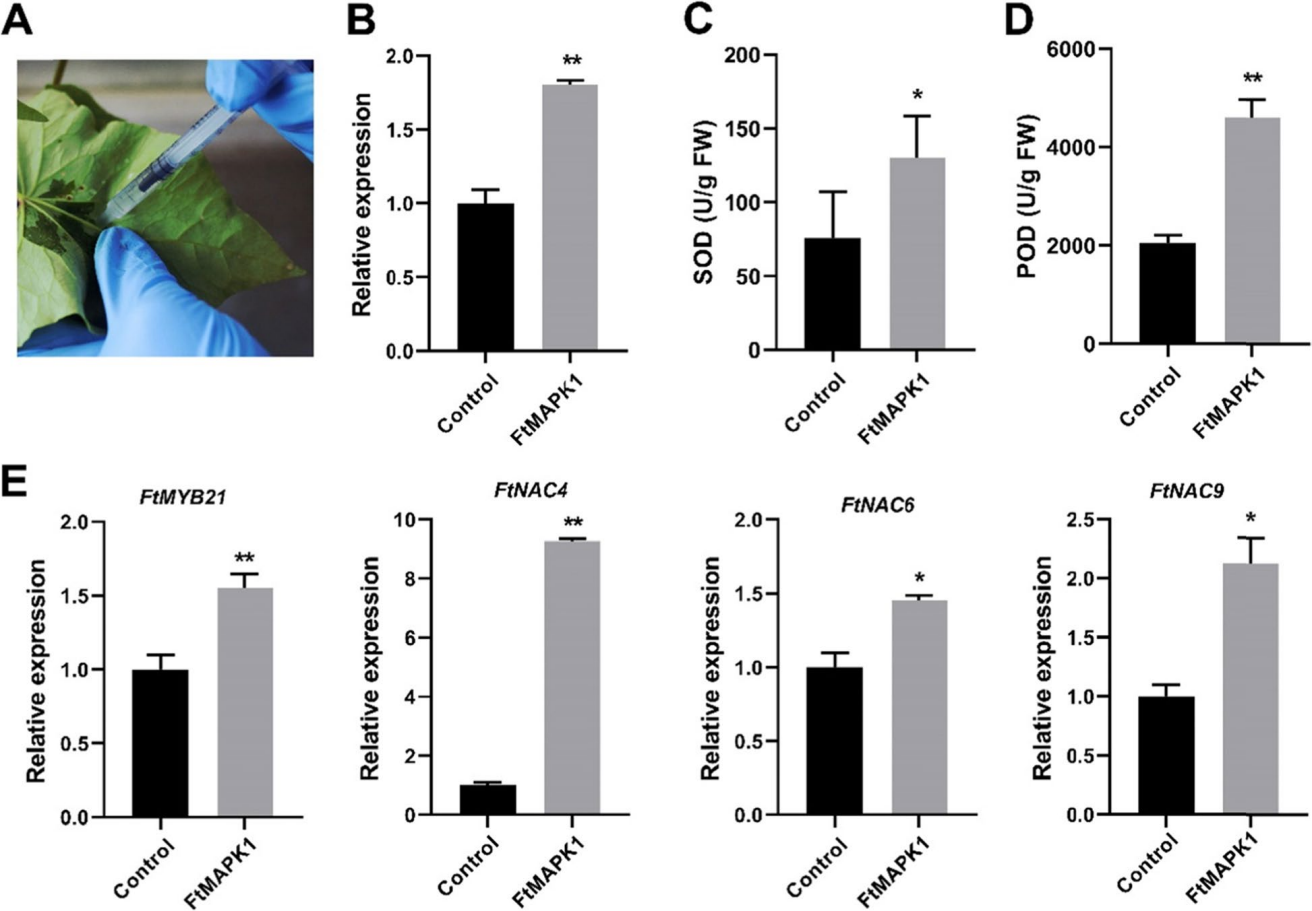
The effect of transient expression of *FtMAPK1* on the antioxidative enzymes activity and the expression of response genes in Tartary buckwheat. **A** Transient transformation of Tartary buckwheat leaves with pCHF3-*FtMAPK1*-YFP and pCHF3-YFP mediated by *Agrobacterium tumefaciens*. **B** Relative expression of *FtMAPK1* after transient expression. **C-D** Activity determination of SOD and POD under abiotic stress. **E** Expression analysis of downstream response genes of *FtMAPK1* after salt treatment. The bars represent the standard deviations of three independent experiments. The asterisks indicate significant differences (*P< 0.05; **P< 0.01)

To determine the effect of *FtMAPK1* overexpression on plant response to salt stress, 4-week-old Tartary buckwheat leaves were tested under the same growth conditions (Fig. 13A). Three days after transient transformation *FtMAPK1*, the expression level of *FtMAPK1* was 1.8 times that of the control group (Fig. 13B). Antioxidant enzymes activity determination showed that the content of SOD and POD in the experimental group were significantly higher than those in the control group (Fig. 13C-D). It indicated that overexpression of *FtMAPK1* enhanced the tolerance of Tartary buckwheat to salt stress.

To further study the mechanism by which *FtMAPK1* regulates the downstream stress-related genes at the transcriptional level, the expression of four stress responsive genes were measured by qPCR (Fig. 13E). The results of the study showed that after salt treatment for 24 hours, the expression levels of *FtMYB21*, *FtNAC4*, *FtNAC6* and *FtNAC9* in the experimental group were significantly induced compared with the control group. Our results suggest that *FtMAPK1* may enhance the resistance to salt stress by increasing downstream stress-related gene expression.

## Discussion

The expansion of *MAPK* family members is indispensable to plant evolution. Compared with the number of genes in other gene families, the amounts of the *MAPK* gene family are relatively conserved in terms of evolution, but with the identification of *MAPK* an increasing number of plants, we found that the number of *MAPK* genes has also expanded. For example, 16, 16, 17, 21, 25 and 26 *MAPK* genes were identified in *Brachypodium distachyon* [22], tomato [23], tobacco [24], poplar [25], banana and apple [26],respectively, while there were 38 in soybean [27]. Legumes and woody species have more *MAPK* genes. We speculate that leguminous and woody plant species may require more *MAPK* genes to participate in their complex transcriptional regulation. To date, we screened only 16 *FtMAPK* genes in the genome of *Fagopyrum tataricum* compared with the number of *MAPK* genes in soybean (38 members), cotton (28 members) and bread wheat (54 members) (Fig. 1).

The number of *MAPK* gene family members is related to genome size, replication events, and ploidy [28]. Higher plants have more *MAPK* genes than lower plants. However, there are 54 *MAPK* genes in the bread wheat genome. Therefore, many *MAPK* genes are related to their polyploidy in bread wheat [29]. Therefore, we speculate that genome duplication and plant evolution are essential for expanding *MAPK* gene family members.

Researchers analysing the *MAPK* genes of 40 species found that *MAPK*s can be divided into six different subfamilies (A, B, C, D, E, and F) [28]. LIAN et al. [30] conducted phylogenetic tree analysis on 20 *AtMPK*s, 15*TaMAPKs* and 17 *OsMPK*s, and classified them into seven groups (A, B, C, D, E, F, and G). In this study, the A, B, C and D subfamilies containing TEY/TDY conserved motifs were consistent with those previously reported (Fig. 3B), and there were no E or F subfamilies. However, the two subfamilies E and F are shared by lower eukaryotes and gymnosperms, and we speculate that this may also be a reason for the expansion of gene family members. The emergence of new motifs in MAPK led to an expanded diversity of *MAPKs*. MAPK not only contains TEY/TDY motifs but also contain characteristic motifs such as MEY, TEM and TQY. The diversification of distinctive motifs provides ideas for investigating the function of *MAPK*s, and the diversification of functions enables plants to enhance their ability to adapt to the environment. Here, the 16 *FtMAPK*s contained only TEY/TDY characteristic motifs. This result also shows that the motifs in MAPK in Tartary buckwheat are relatively conserved. The increase in the number of *MAPK* subfamilies and the diversification of characteristic motifs provide new ideas for understanding the evolution and gene function of the *MAPK* gene family.

Promoters control the changes in their structure and morphology in different stages of plant growth and development and the interaction between plants and the environment. Throughout the long process of biological evolution, plants have acquired complex gene regulation mechanisms to mitigate the effects of unfavourable environments. *MAPK* promoters include stress-related *cis*-acting elements, including ABA-responsive *cis*-acting (ABREs) and LTRs in chickpea [6]. In addition, the above mentioned *cis*-acting elements were found in the promoters of the *MAPK* genes of *Solanum lycopersicum* [23], *B. distachyon* [22] and cucumber [31], and the expression level of *MAPKs* was changed after stress treatment. In our research, most *FtMAPK*s have *cis*-regulatory elements involved in ABA and LTR responses. Nevertheless, it is worth noting that 5 *FtMAPKs* (*FtMAPK2*, *FtMAPK4*, *FtMAPK9*, *FtMAPK12* and *FtMAPK16*) contain MYB-binding sites involved in drought-induced responses, and 3 *FtMAPK*s (*FtMAPK3*, *FtMAPK10* and *FtMAPK16*) can bind to MYB-binding sites to regulate the expression of flavonoid synthesis genes. Therefore, in-depth research on the structure, function and expression pattern of promoters will help clarify the regulatory mechanism at the transcriptional level of gene expression and enhance understanding of the life process. However, based on the current research status of promoters, the regulatory mechanisms of most promoters at the transcriptional level remain unclear. In the future, it will be necessary to explore the active sites of promoter-related elements through biological experimental techniques (such as CRISPR/Cas9) to study the transcriptional regulation mechanism of downstream target genes.

The *MAPK* cascade signalling pathway is a conserved protein kinase cascade signalling pathway ubiquitous in eukaryotes [32, 33]. Many research results show that the plant *MAPK* cascade signalling pathway can be widely involved in various response signalling pathways such as those pertaining to drought, high salt and other abiotic stresses, various plant hormones and other signalling molecules, and growth and development [34, 35]. *MPK6* is involved in the environmental response process. Under cold treatment, it can enhance cold tolerance and allow plants to survive [36]. After several drought stress treatments were applied to peanut plants, it was found that the expression of *MAPK*s was upregulated in the *MAPK* signalling pathway [37]. In this study, the transcription of *FtMAPK1* was upregulated under hormone, drought and salt treatment, which suggested that this gene might also have important biological functions in abiotic stress and hormone signalling. Notably, the expression level of *FtMAPK4* was downregulated under the above treatment, and it is speculated that the activation of MAPK4 protein kinase activity may not be related to its transcription level.

In this study, *FtMAPK1* and *FtMAPK3* were highly expressed in the roots, stems and leaves; *FtMAPK5* had higher expression levels in the roots, leaves and flowers; and *FtMAPK8* and *FtMAPK10* were highly expressed in the leaves and roots (Fig. 8). In *Camellia sinensis*, *TEA006436.1* is highly expressed in the apical buds, flowers, fruits, mature leaves and stems [38]. In *Gossypium raimondii*, *GrMAPK27* has the highest transcription level in the leaves, and *GrMAPK16* is expressed in all tissues (roots, stems, leaves and petals) and organs (anthers, ovules and fibres) [9].

*MAPK* genes also present tissue-specific expression patterns. In *Triticum aestivum*, *TaMAPK12* and *TaMAPK15* are expressed only in the roots and flowers, respectively [30]. Compared with other tissues, *SlMAPK7* and *SlMAPK12* are highly expressed in stamens [23]. Here, only *FtMAPK12* shows tissue-specific expression in the leaves and flowers of *Fagopyrum tataricum*. *FtMAPK1* and *FtMAPK3* are remarkedly highly expressed in the stems. This indicates that these genes play potential roles in developing leaves and stems in *Fagopyrum tataricum*. These results provide ideas for further research on the biological functions of MAPK genes.

The molecular mechanism of plant stress resistance has been a hot topic in biological research. However, there are relatively few studies on the function of *MAPK*s in response to adversity. It has been reported that *MAPK*s can participate in abiotic stress responses. For instance, upregulated expression of tobacco *MAPK* genes can improve plant resistance to abiotic stress [39]. Overexpression of *OsMAPK5* can positively regulate the tolerance of plants to drought, salt and cold stress [40]. In this study, we aimed to fully understand the function of *FtMAPK* genes in Tartary buckwheat. Correlation analysis has a potential role in the investigation of gene function. We found that most *FtMAPK*s (*FtMAPK1*, *FtMAPK2*, *FtMAPK6*, *FtMAPK7*, *FtMAPK8*, *FtMAPK9*, *FtMAPK10*, *FtMAPK11*, *FtMAPK13* and *FtMAPK15*) were positively correlated with one or more *FtMYB*, *FtNAC*, *FtbZIP* and *FtWRKY* involved in the stress response. We speculate that these *FtMAPK*s may have the same expression pattern as their significantly related transcription factors. This research provides information for research on *FtMAPK*s functions.

## Conclusion

In this study, we identified 16 *FtMAPK* genes from the Tartary buckwheat genome. A phylogenetic tree was constructed showing that all of the MAPKs contain TDY and TEY characteristic motifs. Analysis of all MAPK protein motifs strongly supports this prediction. Correlation analysis showed that *MAPK1* and stress-related transcription factors have the same expression pattern. Additionally, a protein interaction network was constructed, which indicated that several *MAPK*s participate in the regulation of the expression of stress and development-related genes. Salt stress experiment showed that *FtMAPK1* ultimately enhanced the stress resistance of Tartary buckwheat by increasing the activity of antioxidant enzymes and the expression of stress-related genes.

## Materials and methods

### Identification of *FtMAPK* genes

To screen the MAPK genes in Tartary buckwheat, we obtained genomic data from http://www.mbkbase.org/Pinku1/. *Arabidopsis* MAPK cascade proteins from https://www.arabidopsis.org/ were used as queries to search for Tartary buckwheat proteins using BLASTP. Candidate genes were submitted to https://www.ncbi.nlm.nih.gov/cdd to verify their characteristic kinase structural motifs further.

### Analysis of the main characteristics of *MAPK* family members in Tartary buckwheat

The phylogenetic tree of *MAPK* amino acid sequences in Tartary buckwheat and other three species (*Arabidopsis thaliana, Oryza sativa* and *Gossypium raimondii*) was constructed with MEGA 7.0.26 by the neighbour-joining (NJ) method and 1000 bootstrap replications, and the phylogenetic tree was visualized through EvolView (https://evolgenius.info//evolview-v2/#mytrees/1/2). We analysed and visualized the distribution of all *MAPKs* on chromosomes of Tartary buckwheat by TBtools [41]. The phylogenetic tree of Tartary Buckwheat *MAPK* gene was constructed by NJ method and visualized by TBtools. The characteristic motifs of MAPKs were determined by MEME (http://meme-suite.org/tools/meme), the number of motifs was 15, and the order of site distribution was zero or one occurrence per sequence. TBtools analyses the gene structure of all *MAPKs*. In addition, to analyse *cis*-acting in the promoter region of the *FtMAPK* genes, we truncated the 2 kb sequence upstream of the start codon for *FtMAPK* genes, which was analysed via the PlantCARE database (http://bioinformatics.psb.ugent.be/webtools/plantcare/html/). Finally, protein motifs and *cis*-acting elements were visualized by TBtools. The tertiary structure of FtMAPK proteins was predicted by SWISS-MODEL Interactive Workshop (https://www.swissmodel.expasy.org/interactive).

### Analysis of duplication events and collinearity of *FtMAPK*s

The gene duplication events of Tartary buckwheat *MAPK* and collinearity analysis with six other species were assessed and performed, respectively, according to the method of Yao et al. [42].

### Plant growth, abiotic stress treatment

The Tartary buckwheat cultivar ‘Xiqiao 2’ was grown at the experimental base of Sichuan Agricultural University in Ya'an, Sichuan, China, on May 10. For sampling times and sample storage conditions, refer to Yao et al. [42].

The cultivation of Tartary buckwheat seedlings was performed according to the method of Yuan et al. [43] with slight modifications. Hormonal (100 μM MeJA and 100 μM ABA ) and abiotic stress treatments (200 mM NaCl and 20% PEG6000 (w/v)) were performed according to the method of Zhang [9] and Singh [44]. The cotyledons were collected at 0,3,12,24 and 48 h, flash frozen in liquid nitrogen, and then stored at -80°C for further analysis.

### Construction of a protein interaction network

The MAPK protein sequence was aligned to String (http://string-db.org/) for protein–protein interaction prediction. The regulatory network of the other proteins and the MAPK proteins was visualized using Cytoscape (http://www.cytoscape.org/).

### Correlation analysis between MAPK and transcription factors

To predict the function of *FtMAPK*s, functionally identified transcription factors, including MYB (*FtMYB7*, *FtMYB9*, *FtMYB10*, *FtMYB13* and *FtMYB17*) [45], *FtMYB21* [46], *FtWRKY46* [47], NAC (*FtNAC2*, *FtNAC3*, *FtNAC4*, *FtNAC5*, *FtNAC6*, *FtNAC7*, *FtNAC8* and *FtNAC9*) [48], bHLH (*FtbHLH1* [49], *FtbHLH3* [50] and *FtbHLH4* [51]), *FtbZIP5* [52] and *FtbZIP83* [53] were used to analyse the expression pattern of *FtMAPK*s in Tartary buckwheat. Salt stress transcriptome data were obtained from the NCBI Sequence Read Archive (http://www.ncbi.nlm.nih.gov/Traces/sra/) with the accession number PRJNA528524 [54]. Drought stress (20% PEG6000) transcriptome data were generated in-house (unpublished). The correlation analysis between *FtMAPK* genes and transcription factors was calculated using the cor function in the R language with the default Pearson correlation coefficient.

### Transient expression of *FtMAPK1* in Tartary buckwheat leaves

To further study the function of the *MAPK* gene of Tartary buckwheat, we selected *MAPK1* and cloned the complete coding sequence. The open reading frame (ORF) of *FtMAPK1* was PCR-amplified using primers (Additional file 4). Then, the sequence was inserted into the vector, pCHF3-YFP with Clone Express (Vazyme, C112-02). The recombinant plasmid was mediated by *Agrobacterium tumefaciens* to transform Tartary buckwheat leaves. The empty vector pCHF3-YFP was transiently transformed under the same conditions as those used for the negative control.

Three days after transformation, the leaves of the experimental group and the control group were placed on MS plates containing 200mM NaCl for 24h, and then the superoxide dismutase (SOD) and peroxidase (POD) content of the experimental group and the control group were determined. SOD and POD were determined by the previous methods [55].

### Quantitative real-time PCR analysis

Total RNA was extracted by an EASY spin Plant RNA Kit (Aidlab, China). First–strand cDNAs were synthesized using HiScript® III–RT SuperMix for qPCR (Vazyme, China). The qRT–PCR primers (Additional file 5) were designed via primer 3 (https://www.ncbi.nlm.nih.gov/tools/primer-blast/). Refer to Liu et al. [56] for the selection of the internal reference genes. 2xChamQ Universal SYBR qPCR Master Mix (Vazyme, China) was used for qRT–PCR. The amplification program used were as follows: 98°C for 45 s followed by 34 cycles of 98°C for 15 s and 60 °C for 45 s. The correlative expression data were calculated by the 2^-∆∆CT^ method [57].

### Statistical analysis

The experimental data were visualized by GraphPad Prism 8.0, and the significance was analyzed at the 0.05 and 0.01 probability levels by ANOVA in IBM SPSS Statistics 22.0.

## Supplementary Information

**Additional file 1 : Table S1.** List of the 16 MAPK genes identified in this study

**Additional file 2 : Figure S1.** Analysis of characteristic domains of 16 FtMAPK proteins.

**Additional file 3 : Table S2.** *Cis*-acting elements of *FtMAPKs* promoter

**Additional file 4 : Table S3.** Primers used for construct expression vector of FtMAPK1. For expression of recombinant plasmid in Tartary buckwheat using pCHF-YFP vector.

**Additional file 5 : Table S4.** primer sequences of RT-qPCR

## Data Availability

The *FtMAPK* gene sequences in this study are available in the Tartary Buckwheat Genome Project (http://www.mbkbase.org/Pinku1/). The Tartary buckwheat cultivar ‘Xiqiao 2’ materials used in the experiment came were obtained our laboratory. The datasets supporting the conclusions of this article are included with in this article and its additional files.

## Abbreviations

MAPK: Mitogen-activated protein kinase
*Ft*: *Fagopyrum tataricum*
MeJA: Methyl jasmonate
ABA: Abscisic acid
