# Supplementary figures and images for: Genome-wide identification of MAPK gene family members in Fagopyrum tataricum and their expression during development and stress responses
Source: BMC Genomics. 2022 Feb 3;23:96. doi: 10.1186/s12864-022-08293-2 (PMC8815160; doi:10.1186/s12864-022-08293-2)

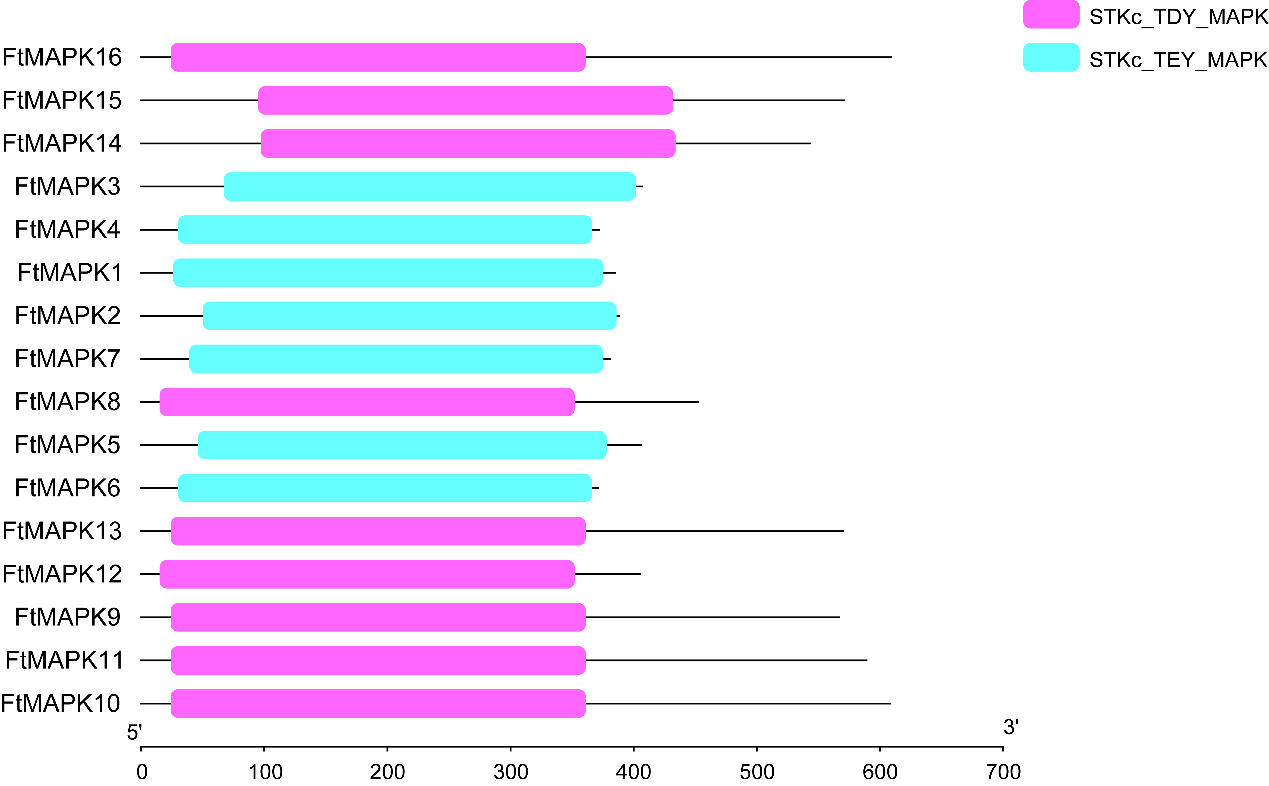


**Fig. S1** Analysis ofcharacteristic domains of 16 FtMAPK proteins.

Supplement: Supplementary file 2 — Additional file 2 : Figure S1. Analysis of characteristic domains of 16 FtMAPK proteins. [file 12864_2022_8293_MOESM2_ESM.docx]
